# President’s Address

**Published:** 1903-01-15

**Authors:** Otto Arnold


### PRESIDENT'S ADDRESS.

BY OTTO ARNOLD, D.D.S.

Read at the Thirty-seventh Annual Meeting of the Ohio State Dental Society, December 2, 1902.

I ask your indulgence for a short time, while I endeavor to fulfill the duties devolving upon the presiding officer of this body, prescribed by a time honored custom, in presenting what you may please to call the annual address.

I greet and welcome you all, to our thirty-seventh annual meeting and trust that it may be fittingly celebrated by both mind and body. We present for your edification a program that is not the product of chance, but which represents the earnest labor and deliberations of the several committees, whom I thank most heartily for their cordial co-operation. It is for you to enjoy. The more you get out of it, the better will its promotors be repaid.

Many of you have come long distances. Some of you have brought valuable contributions for the cause of dentistry, but I daresay that most of us depart from these meetings taking more away than we brought to them. This is as it should be, as it is the very essence of a society's function to benefiit the many rather than the few. An organization that fulfills such a mission need never apologize for its existance.

I regret that a larger percent of Ohio's 2000 dentists do not habitually attend these meetings. Where so many attractions are presented, aimed to satisfy all tastes, it is strange that so many will deny themselves the benefits they would receive from coming into closer touch with each other. Most of us can upon occasion find the time to attend horse races or to take hunting or fishing trips ; but it is remarkable, that a vertible epidemic of dental maladies arise just about the time of the dental meeting, compelling so many against their wills to stay at home. A board of commissioners to solve this problem might not be out of place.

To enumerate the many recent advances made in the art and science of dentistry would be a stupendous task, which I will not now undertake. Since last we met, a new dental law has been enacted which requires all applicants for licenses to practice in Ohio, to be graduates and also to pass an examination of the State Board of Dental Examiners. I believe this to be a long step forward in the direction of higher professional standards and in ultimately bringing about uniform and reciprocal relations with all the states as well. Among other notable events are the Bacteriological and Chemical researches conducted by Drs. Michaels, of Paris and C. E. Kirk, of Philadelphia, from which valuable data have been secured in establishing the cause of dental erosion, also the importance of the saliva as a diagnostic agent in systemic disorders.

In this branch of science, seeking to unravel the mysteries of the unknown, there will always be room for workers, and yet, for obvious reasons this field is neglected, even by those who have the inclination and are fitted for the work. The action recently taken in the New York State Dental Society in raising a liberal fund to encourage scientific research, is highly commendable in stimulating action in this direction. We and others might emulate the example in aiding such an important movement. As an initial step I do therefore recommend, that this society set aside annually one hundred dollars to create a fund to be known as the "scientific research fund." Said fund to be controlled by the directors or a special committee of this body, and to be used only for rewarding original investigators for meritorious discoveries directly related to dentistry. The custodians of the fund to be authorized to co-operate with representatives of other dental organizations having a similar plan of operation, the aim of each being of course the mutual advancement of dental science.

At first thought, some of you may think the above an insignificant amount for such a grand object; but if it were combined with an equal amount set aside by each of twenty-five or more societies, which is not at all improbable, there would be an amount of at least $2,500 available for the purpose annually. I'm sure that such an amount could be spared every year from the natural resources of this society without further tax upon its members, although each would be a contributor by virtue of his membership. Neither would such a plan interfere with the generosity of individuals or societies wishing to contribute independently. I submit this proposition for your respectful consideration.

Such discoveries while of infinite value universally, do more and go farther in establishing a secure footing for dentistry than anything else can, and in answering the arguments of those who oppose dentistry as an independent calling, even to the extent of supplanting the dental by a medical degree. This brings to mind also the proposition that a dentists education should begin in a medical school, about which there is more or less controversy.

The service demanded of the dentist is almost entirely manipulative; calling for the highest order of technical skill for its accomplishment. Indeed, his technical attainments must be *extraordinary;* as all operations upon the teeth must resist physical strain, chemical and vital agencies and possess esthetic and artistic features as well. To properly prepare one to fulfill these requirements a systematic course of manual training must be adopted which best fits the student to this end. This process, to be most efficient must be included in the *beginning* of the course of study along with anatomy, physiology, pathology, chemistry, materia medica and kindred sciences. The dental degree must represent efficiency in all these subjects. If it does, it is here to stay. Our destiny is altogether in the keeping of the dental colleges.

To begin a dentist's education in a medical college, would be detrimental to his manual training. If the medical degree is coveted, a commendable ambition, it should be sought after the dental degree has been obtained. It is in the earlier years that manual skill is best acquired while a matured mind is best fitted for scientific broadening. The remarkable technique of a Paganini or a Paderewski is not acquired late in life, neither can we expect a dental student to be benefitted by deferring his manual training.

I care not how extensive ones knowledge may be in general medicine and surgery or in the allied sciences, if he does not also possess the requisite technical skill to successfully fulfill the demands made upon him in the multitudinous operations required in the mouth, he is a failure as a dentist.

On the other hand, a dentist whose knowledge is confined merely to the technical, is a one sided product; unworthy of the fullest measure of confidence from his co-laborers in other branches of the healing art.

Specialism does not mean narrowness, although it may tend to it. On the contrary, it should stand for thoroughness in the highes sense in whatever branch it is applied. The dentist should possess a sufficiently thorough knowledge in pathology to recognize the various lesions as are here manifested in the mouth, and to discriminate between simple stomatitis, mercurial salivation or syphilitic complications. He should be sufficiently versed in chemistry and bacteriology to test the secretions within the mouth, that he may determine more or less accurately the factors at work in destroying the teeth. All of this, and more, the twentieth century dentist must know and the twentieth century dental colleges must teach.

It is claimed that dental colleges have exalted the technical branches and almost excluded the scientific from their courses of study. If there are such they are not worthy of the name and should be ostracised by the profession. There is no reason why the curricula of dental colleges should not be properly balanced nor that their teachers should be inferior.

I believe in the broadest possible culture of the dentist and can see no reasonable excuse why he should not be the peer in learning of other professional men. The door to knowledge is an open one. Books upon all subjects are to be had almost for the asking. If he thinks he knows it all after graduation, and ceases to be a student of nature and avoids books, which I fear is too often the case—he must not expect equal recognition from those advanced in the scientific and intellectual world. Thus it is largely individual merit that counts after all.

From the celebrated address of Sir Michael Foster, at the meeting of the International Dental Congress, held at Cambridge, England, I quote as follows:

"From the broad basis of a general school education the narrowing of professional training begins." "The dentist is a healer; his business lies with a very small portion of the human frame, but that portion though small, is still human." "The dentist is a surgeon and something more. He has, like the physician, to possess a general knowledge of disease, and like the surgeon a certain skill of hand; but, besides this he has to acquire a special manual dexterity never called for in the surgeon, and to possess a special knowledge of metallurgy, of chemico-physics, and of mechanics of which neither the physician nor the surgeon need know anything at all."

"The dentist if he is to excel in his art, can not hope to know all that the physician and surgeon must know, such being the case, I would therefore urge you to continue on the lines which will train a dentist for his own profession from first to last."

These words coming from such an authority, are the greatest tribute ever paid to the independent system of dental education. A system which originated in this country, a little more than half a century ago, under most discouraging conditions, and whatever its past short comings may have been, it slowly but surely forged ahead, until it has attained a position of importance in the educational affairs of the world.

Before the next annual meeting of this society, the course of study in all dental colleges will have been extended to four years. Surely, this will be a great advance in our educational system, permitting a curriculum so broad, so thorough, that its product must be the equal of any profession.

In importance, in attainments, in dignity, dentistry occupies to-day a position of safety that will endure, so long as its diciples remain true and practice the high principles demanded of professional men. The outlook was never brighter. Let us always be loyal to its best interests, each contributing what he can to exalt its standards. As healers, let us emulate the example of Him who went about doing good to his fellowmen, and all will be well.

